# Obituary—Professor Paolo Pelosi, President Elect of SIAARTI

**DOI:** 10.1186/s44158-023-00103-9

**Published:** 2023-06-12

**Authors:** Lorenzo Ball, Chiara Robba

**Affiliations:** 1 Department of Surgical Sciences and Integrated Diagnostics (DISC), University of Genoa, Genoa, Italy; 2 Anesthesia and Intensive Care, San Martino Policlinico Hospital, IRCCS for Oncology and Neurosciences, Genoa, Italy

Paolo Pelosi, a giant in the field of Anesthesia and Intensive Care and President-elect of the Italian Society of Anesthesia, Analgesia and Intensive Care Medicine (SIAARTI), passed away on May 30, 2023.

We will miss him, and have special thoughts for his family.

He was a friend and colleague and dedicated his whole life to teaching and research, with a continuous and enthusiastic activity for the advancement of science.

He was also a strong supporter of our National Society official Journal (Journal of Anesthesia, Analgesia and Critical Care - JAACC) and we therefore decided to publish this obituary from two strict Paolo collaborators, Drs Chiara Robba and Lorenzo Ball. **Giorgio Conti, MD (JAACC Editor in Chief)**

**Fig. 1:**
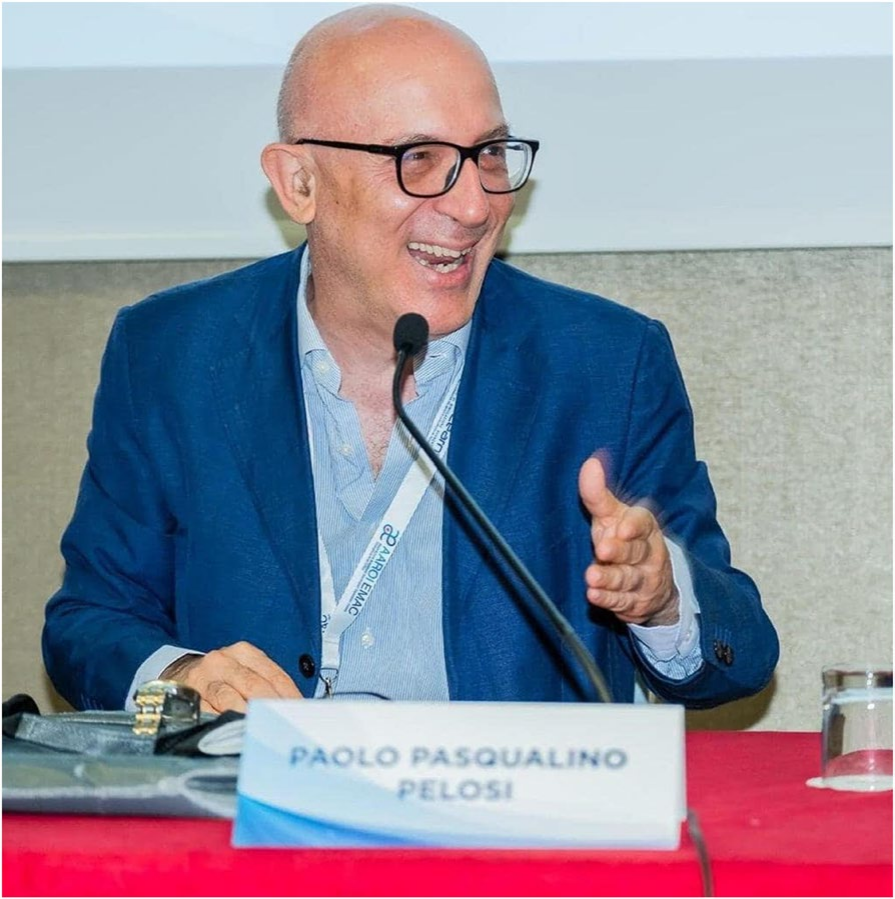
Professor Paolo Pelosi

Professor Paolo Pelosi, MD, FERS, FESAIC (Fig. 1), among the most brilliant and inspiring leaders in the field of anesthesia and intensive care medicine, died at 60 years of age on May 30th 2023. This news shocked the scientific and clinical community in Italy and around the world, where he was universally recognized as one of the greatest experts in the field of mechanical ventilation, a topic that he developed throughout his long career both in the operating room and in the intensive care setting.

As active member of national and international societies, as past president of the European Society of Anesthesia and Intensive Care (ESAIC) and as president elect of the Italian Society of Anesthesia, Intensive Care and Pain Medicine (SIAARTI), he has promoted and supported the unity of our discipline in its different branches, he contributed enormously to the understanding of the pathophysiology of respiratory failure and was a pioneer in translating to the operating theatre the concepts of protective ventilation. As co-founder and chair of the protective ventilation network (PROVEnet), he contributed to the conception and execution of several large, randomized trials in the field of mechanical ventilation in the operating room and in intensive care, boosting the role of evidence-based medicine in our discipline.

During the COVID-19 pandemics, he was among the first intensivists in Europe, together with other colleagues from northern Italy, dealing with the care of critically ill patients with severe COVID-19 pneumonia. In this context, he did not only contribute to the scientific research in the field, but he passionately shared his thoughts, intuitions and clinical experience with colleagues around the globe through countless online conferences, and with the general audience with several interviews in topnotch international broadcast networks, in a phase when the whole world was looking at Italy to understand how this disease was impacting our society.

Professor Pelosi was a demanding and stimulating research chief, but always open to discussion and unceasingly keen to motivate his team in carrying on both physiological and clinical studies with the final aim of improving the quality of care of our patients. His path ended prematurely, and we will not have the chance of having him as President of the SIAARTI. His main declared goal was to further enhance the ability of SIAARTI to act as a tool for his members to support and promote clinical research, an ambitious plan that will be carried over by the Society.

Many of us have lost not only an extraordinary scientist and a reference in the discipline, but a mentor and a friend. His lessons will live in the daily professional life of the colleagues that had the chance to work with such a uniquely brilliant mind, especially the young researchers and clinicians that he has inspired.

We feel privileged to have had the opportunity to work with you.

Thank you and goodbye Professor Pelosi, and as you always said to us, “to the next”. We will miss you.

